# RNA Splicing Is Responsive to MBNL1 Dose

**DOI:** 10.1371/journal.pone.0048825

**Published:** 2012-11-15

**Authors:** Sonali P. Jog, Sharan Paul, Warunee Dansithong, Stephanie Tring, Lucio Comai, Sita Reddy

**Affiliations:** 1 Department of Biochemistry and Molecular Biology, Institute for Genetic Medicine, University of Southern California, Los Angeles, California, United States of America; 2 Department Molecular Microbiology and Immunology, Institute for Genetic Medicine, University of Southern California, Los Angeles, California, United States of America; University of Texas MD Anderson Cancer Center, United States of America

## Abstract

Myotonic dystrophy (DM1) is a highly variable, multi-system disorder resulting from the expansion of an untranslated CTG tract in *DMPK*. In DM1 expanded CUG repeat RNAs form hairpin secondary structures that bind and aberrantly sequester the RNA splice regulator, MBNL1. RNA splice defects resulting as a consequence of MBNL1 depletion have been shown to play a key role in the development of DM1 pathology. In patient populations, both the number and severity of DM1 symptoms increase broadly as a function of CTG tract length. However significant variability in the DM1 phenotype is observed in patients encoding similar CTG repeat numbers. Here we demonstrate that a gradual decrease in MBNL1 levels results both in the expansion of the repertoire of splice defects and an increase in the severity of the splice alterations. Thus, MBNL1 loss does not have an all or none outcome but rather shows a graded effect on the number and severity of the ensuing splice defects. Our results suggest that once a critical threshold is reached, relatively small dose variations of free MBNL1 levels, which may reflect modest changes in the size of the CUG tract or the extent of hairpin secondary structure formation, can significantly alter the number and severity of splice abnormalities and thus contribute to the phenotype variability observed in DM1 patients.

## Introduction

Myotonic dystrophy (DM1) is a multi-system disorder, demonstrating myotonia, skeletal muscle weakness and wasting, cardiac arrhythmias, CNS dysfunction, cataracts, insulin resistance and endocrine disorders [1]. DM1 is a highly variable disorder with patients demonstrating a wide range of symptoms and severity. Disease pathology demonstrates inter-generational variability or genetic anticipation, which manifests generally as an increase in the number and severity of symptoms in successive generations within a pedigree [1]. The genetic basis for DM1 is the expansion of a CTG tract in the 3′untranslated region of *DMPK* [2]. CTG repeat expansions frequently occur in successive generations and as the repeat tract size broadly correlates with disease severity, intergenerational repeat expansion is believed to underlie genetic anticipation [3], [4]. It is of interest to note however that phenotype variability is observed in individuals with similar tract lengths [4]. In this set of experiments, molecular deficits, which occur downstream of CTG repeat expansion that contribute to DM1 disease variability are examined.

Expanded CUG repeat RNA form metastable, slippery hairpin secondary structures *in vitro* and the stem of such hairpins binds the splice regulator, Muscleblind-like 1 (MBNL1) [5], [6], [7]. Significantly, cross-linking experiments demonstrate that MBNL1 binding to double strand CUG RNA increases as a function of the number of CUG repeats [6]. In other studies computer predicted secondary structures of the *DMPK* 3′UTR with expanded CUG repeats show either the formation of a single large hairpin or two or more shorter hairpins with similar calculated free energy [5]. Although the structure of CUG repeat sequences *in vivo* is unknown, hairpin formation is strongly supported by the fact that expanded CUG repeat encoding RNAs bind and aberrantly sequester MBNL1 to form CUG-protein aggregates or foci within the nucleus [6], [8]. Such aggregate formation serves to decrease free MBNL1 levels and can as a consequence result in the mis-splicing of MBNL1 target RNAs [9].

DM1 pathology is widely regarded to result from the aberrant splicing of a set of physiologically important RNAs [10]. For example, aberrant splicing of the chloride channel and the insulin receptor RNAs has been shown to play a causal role in the development of myotonia and insulin resistance, respectively in DM1 [11]–[13]. Significantly, Mbnl1 loss in mice recapitulates key features of DM1 pathology and can account for more than 80% of the splice defects observed in DM1 mouse models expressing expanded CTG tracts [14], [15]. Taken together these data demonstrate that MBNL1 loss plays a central role in the manifestation of DM1 pathology.

Based on these data we hypothesized that dose variations in free MBNL1 levels could play an important role in disease variability in the DM1 patient population. Using human myoblasts as a model system we show that MBNL1 depletion does not show an all or none effect but rather demonstrates that incremental depletion of MBNL1 results in an increase both in the number and severity of splice defects. Our results therefore support the hypothesis that variations in free MBNL1 levels, which may occur as a function of the size of the CUG tract length expansions or the extent of hairpin secondary structure formation, play an important role in modulating the number and severity of DM1 symptoms.

## Results and Discussion

**Figure 1:**
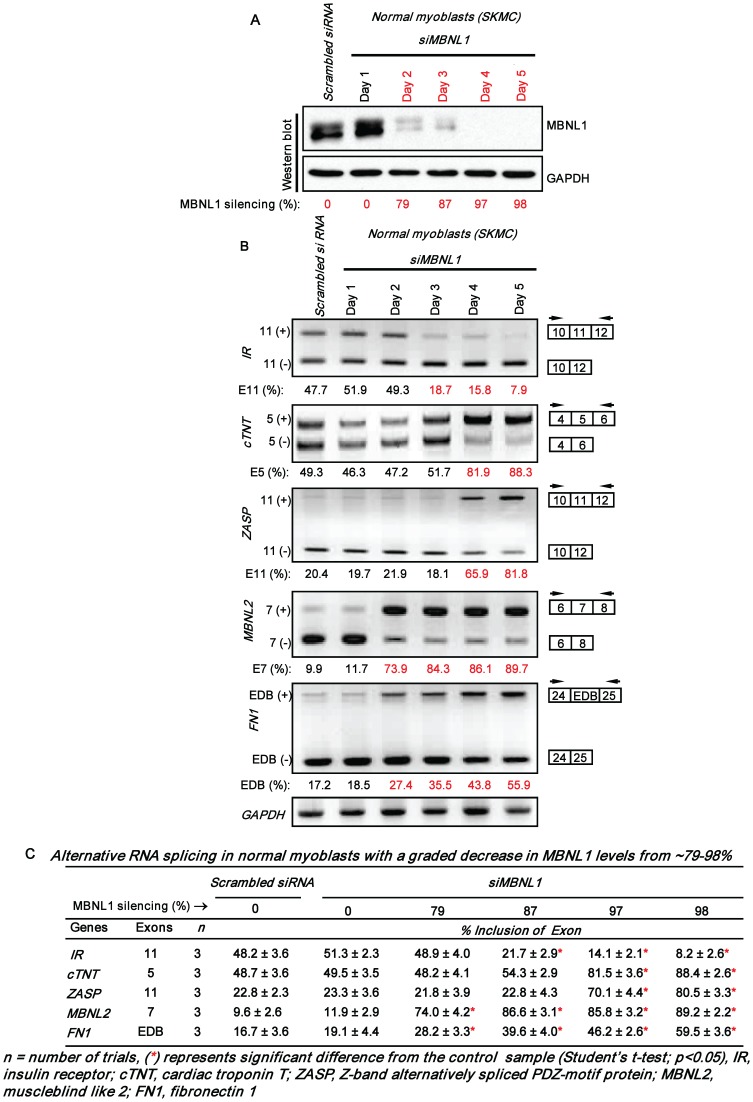
Number and severity of splice defects increase when MBNL1 is silenced incrementally from ∼79% to ∼98%. SkMC were transfected with siRNAs directed against MBNL1 and cell samples on each subsequent day post-siRNA transfection for a period of 5 days, were divided into 4 aliquots where one aliquot was used to measure MBNL1 levels and total RNA was extracted from each of the three other aliquots. Scrambled siRNA transfected samples were harvested on Day 5, the last time point of the experiment. **(A)** Total protein (10 µg) was analyzed by western blot to measure the silencing achieved for MBNL1 at 24 h intervals for 5 days. Blots were probed for GAPDH as an internal control. **(B)** Synthesized cDNAs were subjected to PCR analysis to study RNA splicing as indicated with *GAPDH* RNA as an internal control. In each case the levels of exon inclusion obtained in the experiment shown are indicated. **(C)** The results of RNA splicing as a function of MBNL1 levels in SkMC are tabulated.

**Figure 2:**
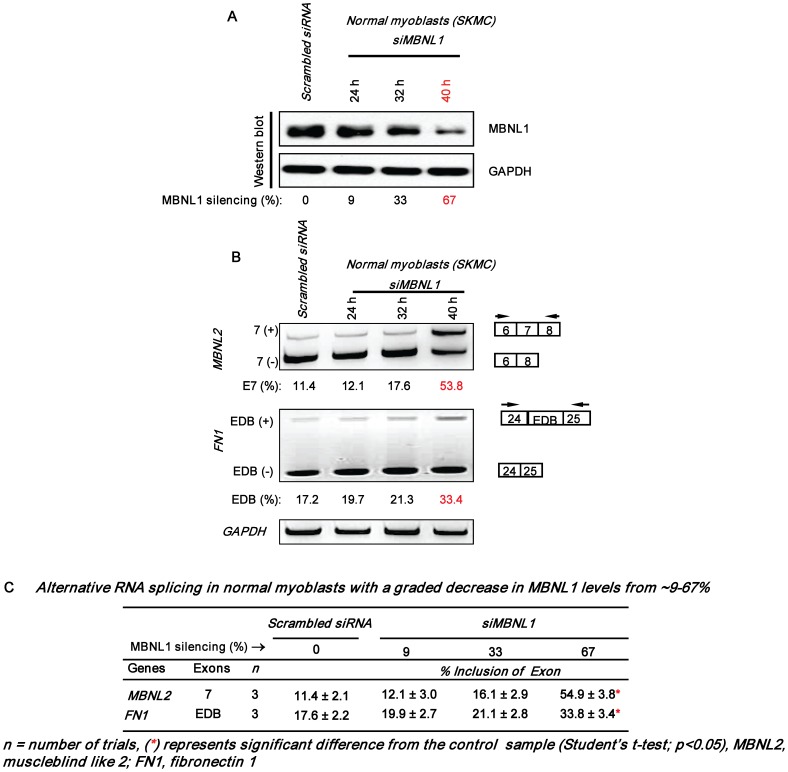
Splice defects observed at ∼67% MBNL1 silencing. SkMC were transfected with siRNAs directed against MBNL1 and cell samples collected 24 h, 32 h and 40 h post-siRNA transfection were divided into 4 aliquots. One aliquot was used to measure MBNL1 levels and total RNA was extracted from each of the three other aliquots. Scrambled siRNA transfected samples were harvested at ∼48 h. **(A)** siRNA mediated down-regulation of MBNL1 at 24 h, 32 h and 40 h in SkMC is shown. Blots were probed for GAPDH as an internal control. **(B)** Synthesized cDNAs were subjected to PCR analysis as indicated with *GAPDH* RNA as an internal control. In each case the levels of exon inclusion obtained in the experiment shown are indicated. **(C)** The results of RNA splicing as a function of MBNL1 levels in SkMC are tabulated.

**Figure 3:**
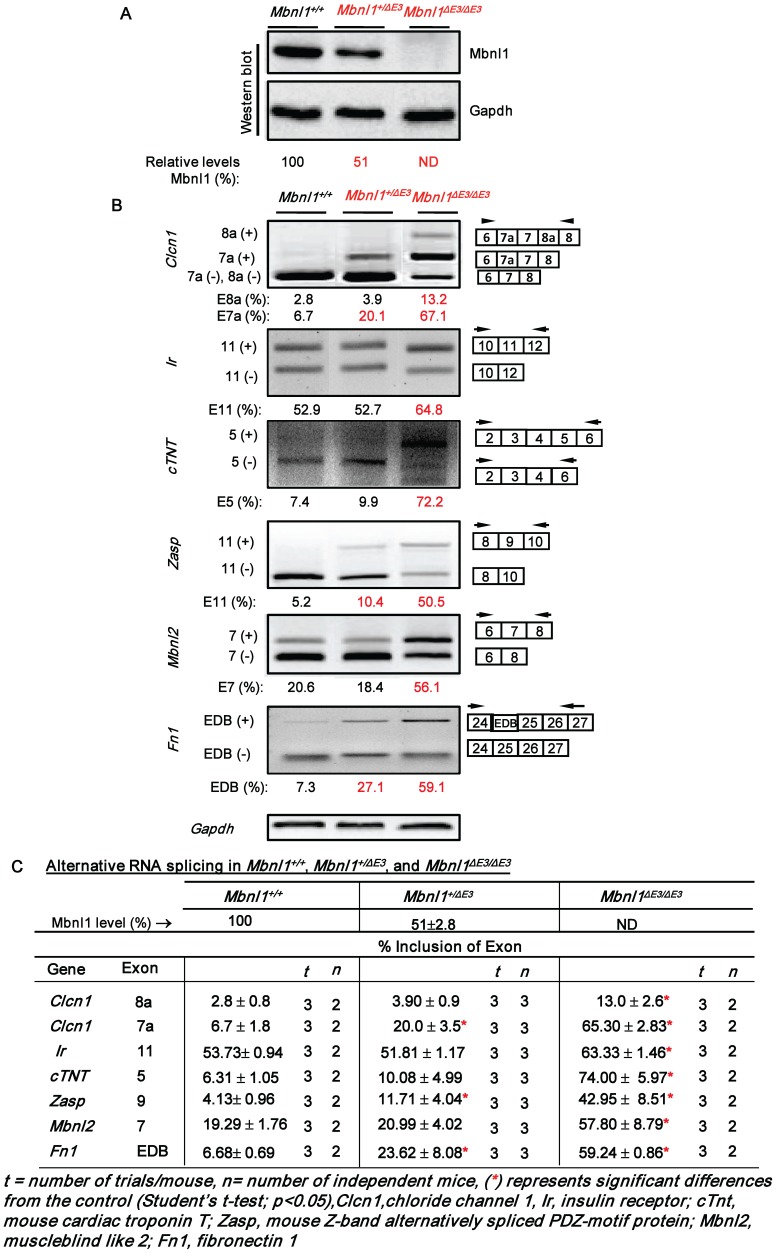
Splice defects in *Mbnl1^+/ΔE3^* and *Mbnl1^ΔE3/ΔE3^* skeletal muscle. Lower limb skeletal muscles from adult wild-type, *Mbnl1^+/ΔE3^* and *Mbnl1^ΔE3/ΔE3^* mice were harvested and divided into 2 aliquots. One aliquot was used to measure Mbnl1 levels and the other aliquot was used study RNA splicing. **(A)** Western blot analysis of steady-state Mbnl1 levels in skeletal muscle of wild-type, *Mbnl1^+/ΔE3^* and *Mbnl1^ΔE3/ΔE3^* mice are shown with Gapdh as an internal loading control. **(B)** cDNAs synthesized from skeletal muscle of wild-type, *Mbnl1^+/ΔE3^* and *Mbnl1^ΔE3/ΔE3^* mice were subjected to PCR analysis as indicated with *Gapdh* RNA as an internal control. In each case the levels of exon inclusion obtained in the experiment shown are indicated. **(C)** The results of RNA splicing examined as a function of Mbnl1 levels are tabulated.

To examine the hypothesis that incremental loss of MBNL1 is a significant contributing factor to the number and severity of DM1 splice defects, we studied the splicing of 5 sample RNAs: insulin receptor (*IR*), cardiac troponin T (*cTNT*), Z-band alternatively spliced PDZ-motif protein (*ZASP*), fibronectin 1 (*FN1*) and muscleblind like 2 (*MBNL2*) that are known to be abnormally spliced in DM1, in normal human myoblasts (SkMC) [9], [11]–[13]. Specifically, MBNL1 levels were incrementally decreased in SkMC by using the cognate siRNAs and the severity of the splice defects were examined. MBNL1 levels were found to decrease by ∼79%, ∼87%, ∼97% and ∼98% at 2, 3, 4 and 5 days post-siRNA transfection in SkMC (Figure 1A). To capture decreases in MBNL1 below 79%, we measured MBNL1 levels at 24, 32 and 40 h post-siRNA transfection (Figure 2A). At these times MBNL1 levels decreased by ∼9%, ∼33% and ∼67% respectively. To test if Mbnl1 dose dependent effects on splicing are observed in skeletal muscle, we tested the splice pattern of the chloride channel (*Clcn1)* RNA in addition to the 5 sample RNAs detailed above in wild-type, *Mbnl1^+/Δ3^* and *Mbnl1^ Δ3/Δ3^* mouse skeletal muscle tissue [14] (Figure 3A, B and C). The *CLCN1* RNAs were not examined in human myoblasts as this RNA is not expressed at significant levels in this cell type (data not shown).

In SkMC, a decrease in MBNL1 levels to ∼67% results in splice defects in two of five RNAs tested, namely, *MBNL2* and *FN1* (Figure 2A, B and C; Figure S1). A further decrease in MBNL1 levels to ∼87% results in splice abnormalities in *MBNL2*, *FN1* and *IR* RNAs (Figure 1A, B and C). At ∼97–98% decrease in MBNL1 levels, all five RNAs, *MBNL2*, *FN1*, *IR*, *ZASP* and *cTNT* show altered splicing (Figure 1A, B and C). Furthermore, the RNAs tested demonstrate an increase in the severity of the splice alterations as a function of decreasing MBNL1 levels. Specifically, in SkMC, for the *MBNL2* RNA inclusion of exon 7 increases from ∼55 to ∼89% (Scrambled Control: ∼10–11%) as the silencing of MBNL1 increases from ∼67% to ∼98%. In the case of *FN1*, inclusion of the EDB exon increases from ∼34% to ∼60% (Scrambled Control: ∼17–18%) as MBNL1 silencing increases from ∼67% to ∼98%. Similarly, inclusion of *IR* exon 11 decreases from ∼22% to ∼8% (Scrambled Control: ∼48%) when MBNL1 silencing increases from ∼87% to ∼98%. Lastly, *cTNT* exon 5 inclusion increases from ∼82% to ∼88% (Scrambled Control: ∼49%) and *ZASP* exon 11 inclusion increases from ∼70 to ∼81% (Scrambled Control: ∼23%) when MBNL1 decreases from ∼97% to ∼98% (Figure 1 and 2). Consistent with the increase in both the number of RNA targets that are mis-spliced and the splice defect severity that results as a function of decreasing MBNL1 levels in normal human myoblasts, Mbnl1 dose dependent effects on RNA splicing were also observed in adult mouse skeletal muscle. Specifically, as previously reported *Clcn1*, *Zasp*, *Fn1, Ir*, *cTnt* and *Mbnl2* RNAs are aberrantly spliced in *Mbnl1^ Δ3/Δ3^* muscle. However, a ∼50% reduction in Mbnl1 levels in *Mbnl1^+/Δ3^* mouse skeletal muscle tissue results in significant *Clcn1*, *Zasp* and *Fn1* RNA splice errors (Figure 3), which are less severe than those observed in *Mbnl1^ Δ3/Δ3^* muscle. Specifically, when Mbnl1 levels decrease from ∼50% to ∼100%, inclusion of *Clcn1* exon 7a increases from ∼20% to ∼65% (wild-type: ∼7%), inclusion of exon 9 of *Zasp* increases from ∼12% to ∼43% (wild-type: ∼4%) and inclusion of the EDB exon in *Fn1* increases from ∼24% to ∼59% (wild-type: ∼7%). No significant splice defects in *Ir*, *cTnt* and *Mbnl2* were observed in *Mbnl1^+/Δ3^* mouse muscle (Figure 3). Wild-type, *Mbnl1^ Δ3/Δ3^* and *Mbnl1^+/Δ3^* mice used in this study were on a 129sv background.

**Figure 4:**
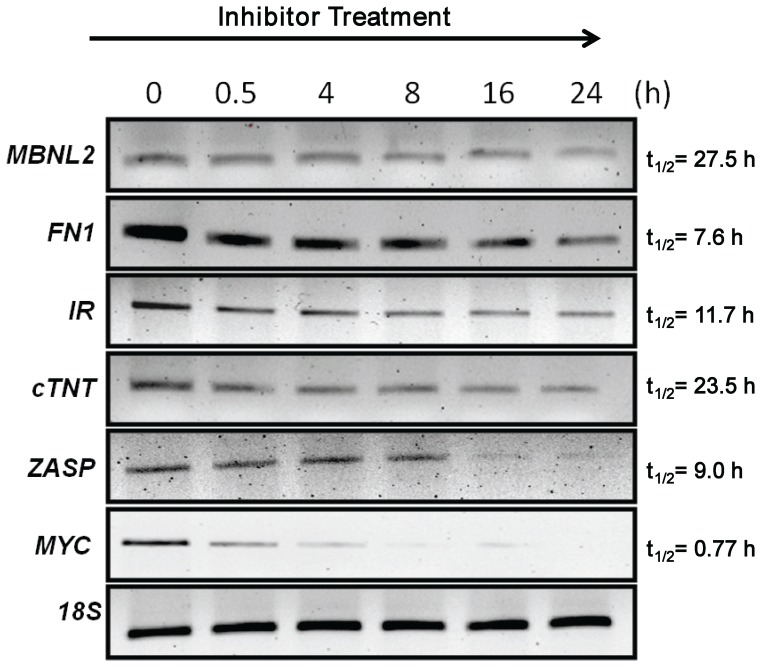
RNA half-life measurements in SkMC. Normal myoblasts were treated with a combination of actinomycin-D and α-aminitin to inhibit transcription. Myoblasts were harvested at different time-points after treatment (0, 0.5, 4, 8, 16 and 24 h) and RNA was extracted. Synthesized cDNAs were subjected to RT-PCR analysis to measure RNA half-lives as previously described (17,18). *MYC*, a short-lived RNA and the long-lived 18S RNA were used as controls. Graphical representation of the average percent of RNA plotted against time from two independent experiments is shown in Figure S2.

**Figure 5:**
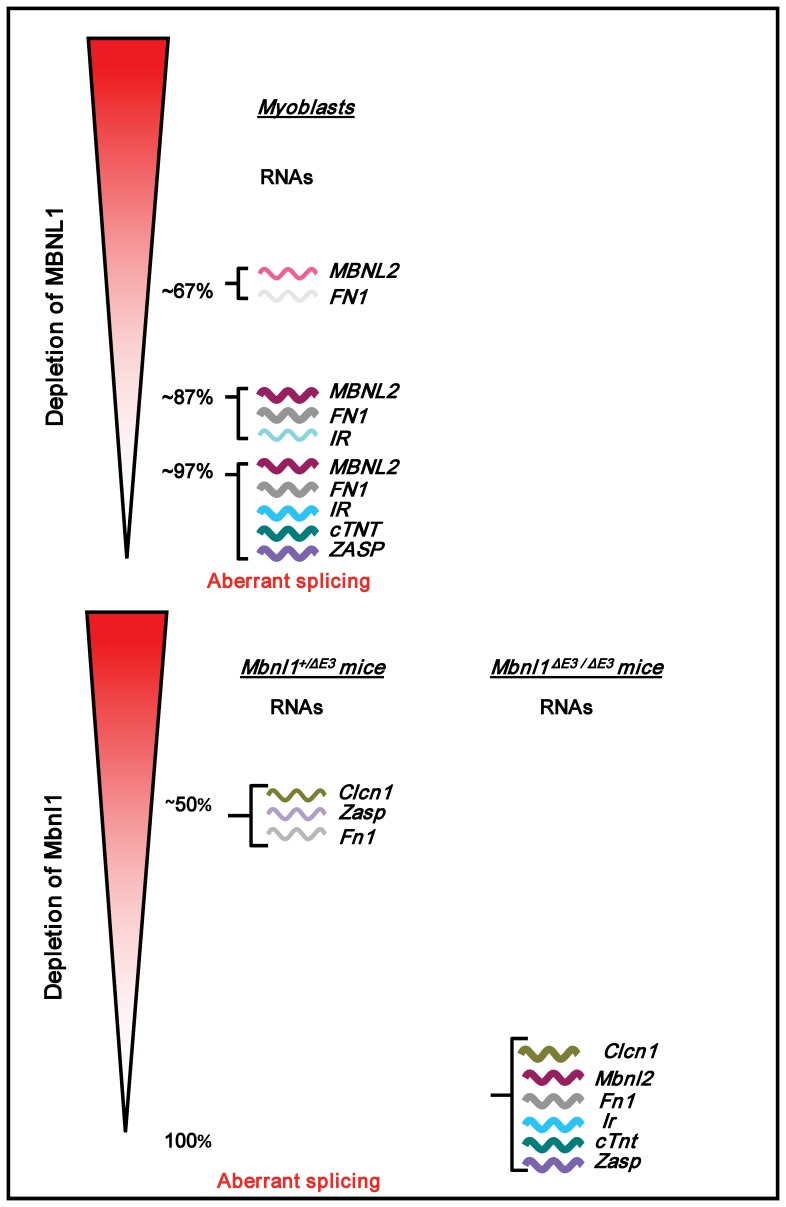
Incremental depletion of MBNL1 results in an increase of both the number and severity of RNA splice defects. RNA splice defects that manifest with the depletion of MBNL1 in SkMC and in *Mbnl1^+/ΔE3^* and *Mbnl1^ΔE3/ΔE3^* skeletal muscle are shown. Line thickness represents the severity of the splice defect.

In other experiments we determined the half-life of each of the RNAs tested in SkMC (Figure 4; Figure S2). A combination of actinomycin D and α-aminitin was used to inhibit transcription in SkMC myoblasts. *MYC*, a short lived RNA and the 18S RNA, which is long lived [16], were used as controls. The half-lives of the 5 RNAs tested in SkMC varied from ∼8–28 h. Half-life measurements were carried out using previously described methods [17], [18]. No overt correlation with the timing of the splice defect detection with the RNA half-lives was observed. Specifically, we did not observe RNAs with longer half-lives demonstrating aberrant splicing at later time points and *vice versa*. Thus, the timing of splice site detection did not correlate with transcript turnover. It is possible that the MBNL1 dose required to elicit splice defects in individual target RNAs reflects differences in ancillary proteins and RNA binding sites, flanking sequences and their secondary structures that influence MBNL1 binding parameters. Alterations in such features maybe responsible for the different MBNL1 doses required to elicit *ZASP* and *FN1* splice errors in human myoblasts and mouse muscles (Figure 5).

Taken together these experiments demonstrate that the spectrum of RNAs that are aberrantly spliced increase as a function of decreasing MBNL1 levels. Furthermore, the data show an increase in the severity of the splice defects as MBNL1 levels decrease (Figure 5). Importantly, these studies demonstrate that once a critical threshold is reached, relatively small changes in MBNL1 levels significantly impact splice outcomes. Therefore, our results support the hypothesis that incremental loss of free MBNL1 can serve to increase both the number and severity of splice defects in DM1.

If the proportional binding of MBNL1 that occurs as a function of the CUG repeat number *in vitro* holds good *in vivo*, the sensitivity of RNA splicing to MBNL1 dosage would predict an increase both in the number of symptoms and their severity when intergenerational CTG repeat expansions occur with successive generations or with somatic expansions in individual patients. Consistent with this model, such increases are documented in flies encoding 60 and 480 CTG repeats [19]. However, variability in the DM1 phenotype, observed with similar but not identical repeat tract sizes may reflect the significant alterations in RNA splice defects that occur with relatively small changes in MBNL1 levels. Such differences may be a consequence of alterations in secondary structure that affect MBNL1 binding or reflect the levels and functions of proteins such as p68/DDX5 that modulate the ability of MBNL1 to bind to the CUG repeat tract that are predicted to act as modifiers contributing to phenotype variability in the DM1 patient population. Lastly, we speculate that a gradual accumulation of MBNL1 in CUG foci, which may occur with time in differentiated cells when compared to rapidly dividing cells, could potentially provide an explanation for the selective sensitivity of terminally differentiated tissues such as skeletal muscle, heart and the CNS, to the progressive toxic effects resulting from expanded CUG repeat RNAs.

## Materials and Methods

### Ethics Statement

All experiments were performed in accordance with the institutional guidelines of the University of Southern California, Los Angeles. The protocol was approved by the Institutional Animal Care and Use Committee at the University of Southern California, Los Angeles (Protocol number: 10347).

### siRNAs

siRNA oligonucleotides were synthesized by Dharmacon (USA), deprotected and the complementary strands were annealed. The sequences of the siRNAs used in this study were:

Scrambled: 5′-GCGCGCUUUGUAGGAUUCGdTdT-3′;

MBNL1∶5′-CACUGGAAGUAUGUAGAGAdTdT-3′.

### Cell Culture, Transfection and RNA Half-life Measurements

Myoblasts were cultured and maintained in SKGM media (Lonza Inc., USA) containing 10% fetal bovine serum and 1% penicillin-streptomycin. siRNA-mediated depletion of MBNL1 was carried out using the methods described in Dansithong et al [21]. Briefly, to decrease MBNL1 levels, myoblasts were plated on 10 cm plates overnight and siRNAs at a concentration of 100 nM were transfected using oligofectamine (Invitrogen), according to the manufacturer’s protocol. Post-transfection cells were harvested at various time points to perform RNA splicing assays and to measure the levels of MBNL1. For RNA half-life analysis, normal myoblasts cultured to ∼80% confluency were treated with a combination of actinomycin D (100 µg/ml) (Sigma Chemical Co.) and α-aminitin (10 µg/ml) (Sigma Chemical Co.). Treated cells were harvested at several time points and RNA was extracted. Synthesized cDNAs were used to measure RNA transcript levels by RT-PCR analysis. A short-lived RNA, *MYC* and the long-lived 18S RNA were used as controls. In all cases, the relative band intensities were measured by densitometry analyses and 18S RNA levels were used for normalization. RNA half-lives were calculated using previously described methods [17], [18]. Primers and PCR conditions are described in Table S1.

### RNA Splicing Studies

To examine splice defects as a function of MBNL1 levels, SkMC samples post-siRNA transfection, were divided into 4 parts where one aliquot was used to measure MBNL1 levels and total RNA was extracted from each of the three other aliquots. Total RNA was isolated using the RNAeasy mini kit (Qiagen), according to the manufacturer’s instructions. RT-PCR analyses of splice isoforms was performed as described in Paul et al [22]. Briefly, cDNA was synthesized from 5 µg of total RNA from each of the three aliquots using the cDNA synthesis kit (Amersham Bioscience Inc., USA). PCR was carried out using each of the three independent sets of cDNAs (150 ng) to test *MBNL2, FN1, IR, ZASP* and *cTNT* splicing using primers and PCR conditions described in Table S2.

*Mbnl1^ΔE3/ΔE3^* mice were a gift from Dr. Swanson (University of Florida College of Medicine). Skeletal muscle tissue from the hind limbs was collected from adult (2–3 month) wild-type (n = 2), *Mbnl1^+/ΔE3^* (n = 3) or *Mbnl1^ΔE3/ΔE3^* (n = 2) mice. The samples were subsequently divided into two aliquots. One aliquot was used to measure Mbnl1 levels by western blot analyses and total RNA was prepared from the second aliquot and RNA splice isoforms were examined. From each set of RNAs, three independent sets of cDNAs (150 ng) were prepared and splicing of *Mbnl2, Fn1, Ir, Zasp* and *cTnt* was studied using PCR conditions as described in Table S3. *Clcn1* splicing was examined as described by Kanadia et al [14]. In all cases, the relative band intensities were measured by densitometry analyses using Gene Tool (Syngene Inc., USA).

### Western Blot Analyses

Whole-cell and tissue extracts were prepared and equal amounts of protein (5–10 µg) were separated by SDS–PAGE and transferred onto Hybond P membranes (Amersham Biosciences Inc., USA). After blocking with 5% skim milk in 0.1% Tween-20 in PBS, the membranes were incubated with the primary antibodies for 2 h at room temp or overnight at 4°C. After incubation, the membranes were washed with 0.1% Tween-20 in PBS and incubated with the corresponding secondary antibodies conjugated with HRP. Signals were detected using the ECL plus detection reagents (Amersham Biosciences Inc., USA) according to the manufacturer’s instructions. MB1a monoclonal antibodies at a dilution of 1∶3000 were used to detect MBNL1 [23]. Membranes were probed for GAPDH as a loading control using goat anti-GAPDH (Santa Cruz Inc., catalog # sc-20357) at a dilution of 1∶3000. The secondary antibody dilutions were 1∶8000 for goat anti-mouse IgG-HRP (Sigma Chemical Co., catalog # A2304), and 1∶5000 for donkey anti-goat IgG-HRP (Santa Cruz Inc., catalog # sc-2056). In all cases, the relative band intensities were measured by densitometry analyses using Gene Tool (Syngene Inc., USA).

## Supporting Information

Figure S1 **Alternative RNA splicing in normal myoblasts with a graded decrease in MBNL1 levels from ∼9-67%.** MBNL1 depletion in SkMC cells is shown in Figure 2. RNA splicing was studied using synthesized cDNAs by PCR analysis using primers for *IR, cTNT and ZASP* with *GAPDH* RNA as an internal control. No significant splice errors were observed in *IR*, *cTNT* and *ZASP* when MBNL1 was depleted from ∼9–67%.
(TIF)

Figure S2 **Graphical representation of RNA half-life measurements in SkMC.** The average decrease in RNA levels in two independent experiments at the time points shown were used to calculate half lives using semi-log plots as previously described [16], [17], [18].
(TIF)

Table S1 **Primers for half-life measurements and PCR parameters.**
(PDF)

Table S2 **Primers used for human RNA splicing and PCR parameters.**
(PDF)

Table S3 **Primers used for mouse RNA splicing and PCR parameters.**
(PDF)
