# Supplementary figures and images for: RNA Splicing Is Responsive to MBNL1 Dose
Source: PLoS One. 2012 Nov 15;7(11):e48825. doi: 10.1371/journal.pone.0048825 (PMC3499511; doi:10.1371/journal.pone.0048825)

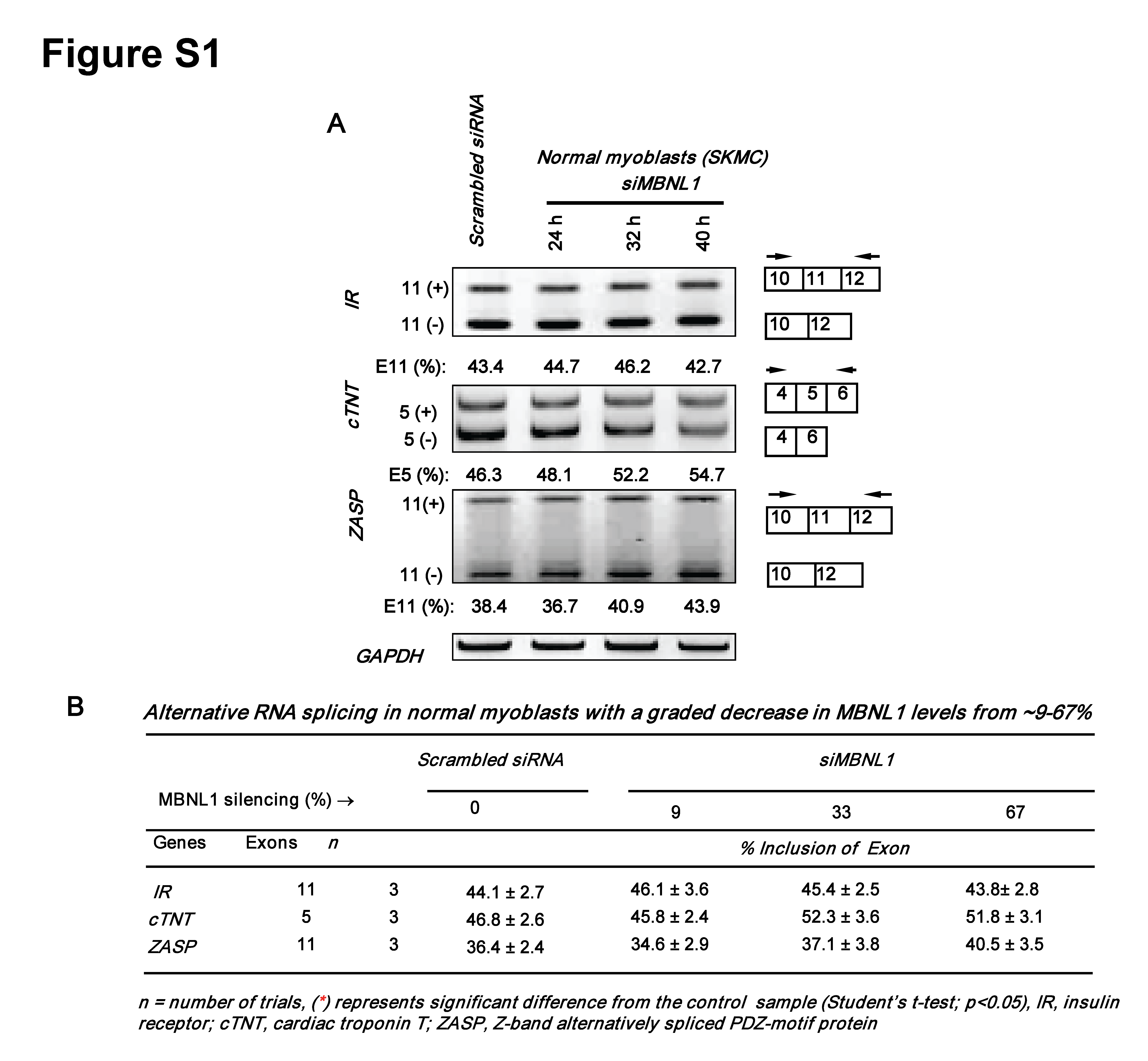

Supplement: Figure S1 — Alternative RNA splicing in normal myoblasts with a graded decrease in MBNL1 levels from ∼9-67%. MBNL1 depletion in SkMC cells is shown in Figure 2. RNA splicing was studied using synthesized cDNAs by PCR analysis using primers for IR, cTNT and ZASP with GAPDH RNA as an internal control. No significant splice errors were observed in IR, cTNT and ZASP when MBNL1 was depleted from ∼9–67%. (TIF) [file pone.0048825.s001.tif]

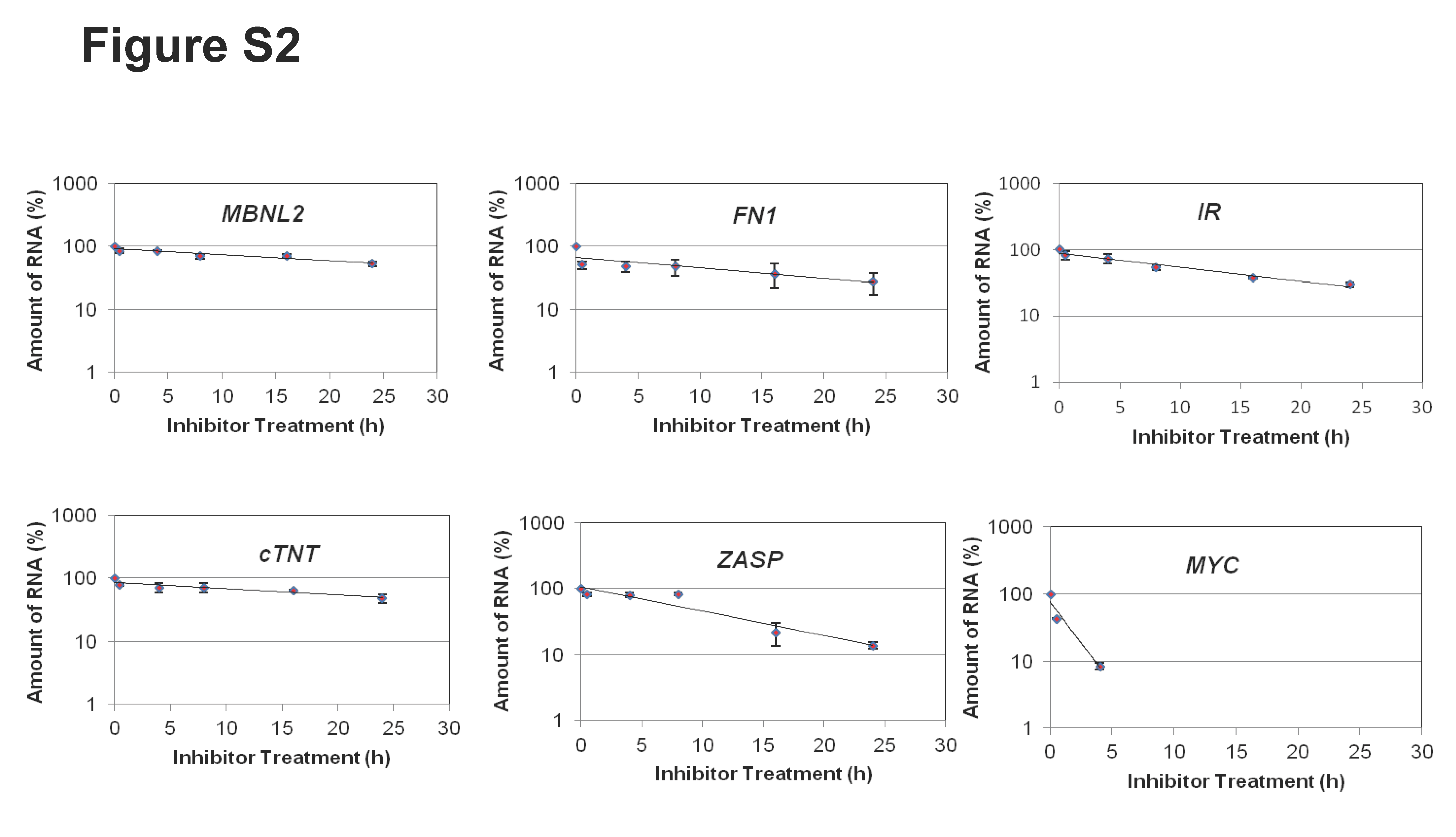

Supplement: Figure S2 — Graphical representation of RNA half-life measurements in SkMC. The average decrease in RNA levels in two independent experiments at the time points shown were used to calculate half lives using semi-log plots as previously described [16], [17], [18]. (TIF) [file pone.0048825.s002.tif]
