# Supplementary material for: RNA Splicing Is Responsive to MBNL1 Dose
Source: PLoS One. 2012 Nov 15;7(11):e48825. doi: 10.1371/journal.pone.0048825 (PMC3499511; doi:10.1371/journal.pone.0048825)
Supplement: Table S1 — Primers for half-life measurements and PCR parameters. (PDF) [file pone.0048825.s003.pdf]

Supplementary Table 1

Primers for half-life measurements and PCR parameters

Primers:	PCR parameters
Insulin receptor (IR)	
**Forward: 5’-CGGAGAGGTGGTGTGTCCCGGCA -3’**<br>**Reverse: 5’-TCTCGAGCCCATAGACCCGGA-3’**	**30 sec at 95^oC, 30 sec at 60^oC, 30 sec at 72^oC for 25 cycles**
Cardiac troponin T (cTNT)	
**Forward: 5’-GCCCAGGTCGTTCATGCCCA-3’**<br>**Reverse: 5’-GCCTCGATCAGCGCCTGCAA-3’**	**30 sec at 95^oC, 30 sec at 60^oC, 30 sec at 72^oC for 25 cycles**
MBNL2	
**Forward: 5’-AAGTGTGTCGGAGGGTGAAG-3’**<br>**Reverse: 5’-CCCCAAAAGTTGTCAGGTTG-3’**	**30 sec at 95^oC, 30 sec at 55^oC, 30 sec at 72^oC for 25 cycles**
FN1	
**Forward: 5’-GGTGACACTTATGAGGGTCCT-3’**<br>**Reverse: 5’-AACATGTAACCACCAGTCTCA-3’**	**30 sec at 95^oC, 30 sec at 55^oC, 30 sec at 72^oC for 25 cycles**
ZASP	
**Forward: 5’-GCAAGGACTTCAACATGCCCCTC-3’**<br>**Reverse: 5’-GTCGTGGAGATGGGATGGGACGC-3’**	**30 sec at 95^oC, 30 sec at 58^oC, 30 sec at 72^oC for 25 cycles**
MYC	
**Forward: 5’-AAGTCCTGCGCCTCGCAA-3’**<br>**Reverse: 5’-GCTGTGGCCTCCAGCAGA-3’**	**30 sec at 95^oC, 30 sec at 58^oC, 30 sec at 72^oC for 25 cycles**
18S	
**Forward: 5’-CGGCGACGACCCATTCGAAC-3’**<br>**Reverse: 5’-GAATCGAACCCTGATTCCCCG-3’**	**30 sec at 95^oC, 30 sec at 58^oC, 30 sec at 72^oC for 25 cycles**
