# Supplementary material for: RNA Splicing Is Responsive to MBNL1 Dose
Source: PLoS One. 2012 Nov 15;7(11):e48825. doi: 10.1371/journal.pone.0048825 (PMC3499511; doi:10.1371/journal.pone.0048825)
Supplement: Table S2 — Primers used for human RNA splicing and PCR parameters. (PDF) [file pone.0048825.s004.pdf]

Supplementary Table 2

Primers used for human RNA splicing and PCR parameters

Primers:	PCR parameters
Insulin receptor (IR)	
**Forward: 5'-CCAAAGACAGACTCTCAGATCC-3'**<br>**Reverse: 5'-ACATTCCCAACATCGCCAAGGG-3'**	**30 sec at 95°C, 30 sec at 60°C, 45 sec at 72°C for 30 cycles**
Cardiac troponin T (cTNT)	
**Forward: 5'-ATAGAAGAGGTGGTGGAAGAGTAC-3'**<br>**Reverse: 5'-GTCTCAGCCTCTGCTTCAGCATCC-3'**	**30 sec at 95°C, 30 sec at 58°C, 45 sec at 72°C for 30 cycles**
MBNL2	
**Forward: 5'-ACAAGTGACAACACCGTAACCG-3'**<br>**Reverse: 5'-TTTGGTAAAGGATGAAGAGCACC-3'**	**30 sec at 95°C, 30 sec at 60°C, 45 sec at 72°C for 30 cycles**
ZASP	
**Forward: 5'-GCAAGACCCTGATGAAGAAGCTC-3'**<br>**Reverse: 5'-GACAGAAGGCCGGATGCTG-3'**	**30 sec at 95°C, 30 sec at 58°C, 45 sec at 72°C for 30 cycles**
FN1	
**Forward: 5'-CATGCTGATCAGAGCTCCTGCAC-3'**<br>**Reverse: 5'-AGGTGAGTAACGCACCAGGAAG-3'**	**30 sec at 95°C, 30 sec at 60°C, 60 sec at 72°C for 30 cycles**
GAPDH human	
**Forward: 5'-TGAAGGTCGGAGTCAACGGATTTGG-3'**<br>**Reverse: 5'-GGAGGCCATGTGGGCCATGAG-3'**	**30 sec at 95°C, 30 sec at 60°C, 60 sec at 72°C for 30 cycles**
