# Supplementary material for: RNA Splicing Is Responsive to MBNL1 Dose
Source: PLoS One. 2012 Nov 15;7(11):e48825. doi: 10.1371/journal.pone.0048825 (PMC3499511; doi:10.1371/journal.pone.0048825)
Supplement: Table S3 — Primers used for mouse RNA splicing and PCR parameters. (PDF) [file pone.0048825.s005.pdf]

Supplementary Table 3

Primers used for mouse RNA splicing and PCR parameters

Primers:	PCR parameters
Clcn1	
Forward: 5'-GGAATACCTCACACTCAAGGCC-3'<br>Reverse: 5'-CACGGAACACAAAGGCACTGAATGT-3'	45 sec at 95°C, 45 sec at 55°C, 45 sec at 72°C for 30 cycles
Insulin receptor (Ir)	
Forward: 5'-GAGGATTACCTGCACAACG-3'<br>Reverse: 5'-CACAATGGTAGAGGAGACG-3'	30 sec at 95°C, 30 sec at 55°C, 45 sec at 72°C for 30 cycles
Cardiac troponin T (cTnt)	
Forward: 5'-GCCGAGGAGGTGGTGGAGGAGTA-3'<br>Reverse: 5'-GTCTCAGCCTCACCCTCAGGCTCA -3'	30 sec at 95°C, 30 sec at 58°C, 45 sec at 72°C for 30 cycles
Mbnl2	
Forward: 5'-CTTTGGTAAGGGATGAAGAGCAC-3'<br>Reverse: 5'-ACCGTAACCGTTTGTATGGATTAC-3'	30 sec at 95°C, 30 sec at 55°C, 45 sec at 72°C for 30 cycles
Zasp	
Forward: 5'-GGAAGATGAGGCTGATGAGTGG-3'<br>Reverse: 5'-TGCTGACAGTGGTAGTGCTCTTTC-3'	30 sec at 95°C, 30 sec at 55°C, 45 sec at 72°C for 30 cycles
Fn1	
Forward: 5'-TGCCCCTATCTCTGATACCG-3'<br>Reverse: 5'-CGATATTGGTGAATCGCAGA-3'	30 sec at 95°C, 30 sec at 55°C, 60 sec at 72°C for 30 cycles
Gapdh	
Forward: 5'-AGAGACGGCCGCCGCATCTTCTTGTG-3'<br>Reverse: 5'-TCTGGGTCCCAGTGATGGCATGG-3'	30 sec at 95°C, 30 sec at 60°C, 60 sec at 72°C for 30 cycles
